# Innovative care models: Expanding nurses’ and optometrists’ roles in ophthalmology

**DOI:** 10.1177/09697330251317670

**Published:** 2025-03-12

**Authors:** Luke Yu Xuan Yeo, Collin Yip Ming Tan, Jemima W. Allen, Charmaine Chai, Khadijah Binte Othman, Yih Chung Tham, Victor Teck Chang Koh, Julian Savulescu

**Affiliations:** Yong Loo Lin School of Medicine, National University of Singapore, Singapore, Singapore; Yong Loo Lin School of Medicine, National University of Singapore, Singapore, Singapore; Department of Paediatrics, Monash University, Clayton, AU-VIC, Australia; Uehiro Oxford Institute, University of Oxford, Oxford, United Kingdom; Yong Loo Lin School of Medicine, National University of Singapore, Singapore; Department of Ophthalmology, National University Hospital, Singapore; Department of Ophthalmology, National University Hospital, Singapore; Department of Ophthalmology, Yong Loo Lin School of Medicine, National University of Singapore, Singapore; Centre for Innovation and Precision Eye Health, Yong Loo Lin School of Medicine, National University of Singapore, Singapore; Singapore Eye Research Institute, Singapore National Eye Centre, Singapore; Ophthalmology and Visual Science Academic Clinical Program, Duke-NUS Medical School, Singapore, Singapore; Department of Ophthalmology, National University Hospital, Singapore, Singapore; Yong Loo Lin School of Medicine, National University of Singapore, Singapore; Yong Loo Lin School of Medicine, National University of Singapore, Singapore; Uehiro Oxford Institute, University of Oxford

**Keywords:** Ophthalmology, allied health, nursing, optometry, physician extenders

## Abstract

The expanding demands of healthcare necessitate novel methods of increasing the supply of trained professionals to enhance the delivery of care services. One means of doing so is to expand allied health professionals’ scope of practice. This paper explores the ethics of two examples of such expansion in ophthalmology, comparing the widely accepted practice of nurses administering intravitreal injections and the relatively less prevalent optometrists functioning as physician extenders. We conducted a literature review of empirical research into both practices and conclude that nurses administering intravitreal injections are ethically justified. With adequate standardized training, optometrists can also function as primary eye care providers to improve accessibility to eye care. We provide an algorithm for the ethical introduction of innovative expanded allied healthcare.

## Introduction

**Figure 1.**
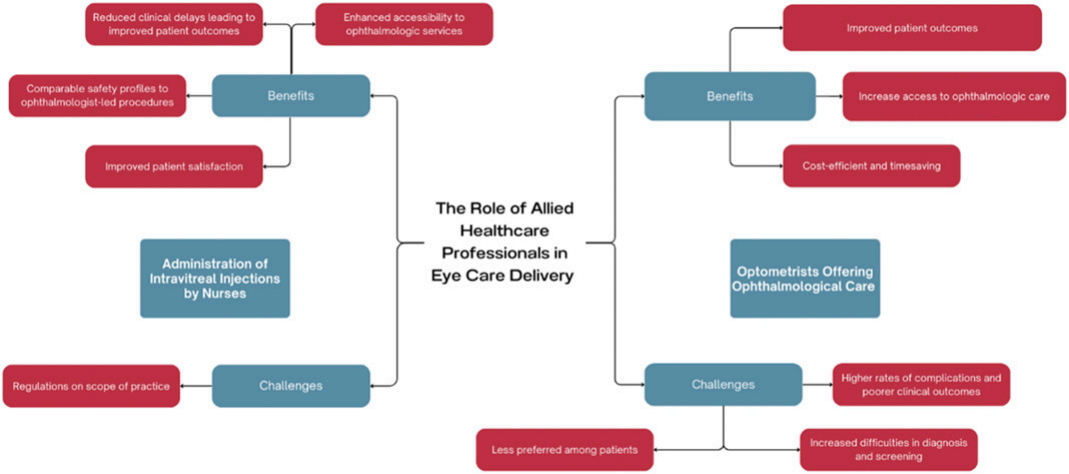
Overview of risks and benefits of nurse and optometrist-led ophthalmological care.

Medical resources, including the provision of professional care, are inevitably limited. However, as population age and possibilities for care increase, there is an increasing gap between what could be provided and what is provided. One way to address this is be expanding the scope of care of allied health professionals. In this paper, we focus on two examples in ophthalmological care: nurses providing intravitreal injections and optometrists acting as physician extenders (see Figure 1).

## Background

The introduction of novel drugs and devices is rigorously regulated. It typically requires randomized controlled trials (RCTs) to demonstrate safety and effectiveness before licensing. However, it may be ethically permissible to administer experimental treatments if ethical requirements are satisfied.^1^ While there are robust requirements for the introduction of treatment variations,^1^ there is a relative dearth of ethical guidance regarding how to responsibly implement variations in medical or surgical practice or expansion of allied health roles. Such variations are often described as innovations, rather than research.^2^ However, it is important to differentiate true “innovation” from routine variations in procedures (i.e., due to unavoidable differences between patients, diseases, and clinical factors).^3^

Unlike experimental treatments, new models of delivering care (i.e., innovative models of allied healthcare) often do not require systematic or rigorous evaluation prior to their use. The starkest example of this is the introduction of artificial intelligence (AI) into clinical care, where rigorous evaluation via an RCT is often not performed.^4^

Given this lack of rigorous evaluation, how should hospitals and healthcare providers decide whether to adopt an innovative model of care?

We propose an ethical framework for evaluating innovative care that can be applied to allied healthcare. This framework considers the following three issues: (1) scientific evaluation of its benefits and harms, (2) cost-effectiveness, and (3) patients’ informed consent.

The first step requires weighing up the expected harms and benefits of a particular innovative model of care. To do this, evaluators should investigate the existing clinical evidence. The expected benefits (or harms) could be evaluated as the product of the scientific probability multiplied by its ethical value. Determining ethical value of expected benefits (or harms) depends on the theory of wellbeing (e.g., hedonism, preference, and objective), but generally this evaluation should not include values which are not commonly held (e.g., values based on religious beliefs).^1^

Depending on the stage of development and testing, there may be varying degrees of confidence in the clinical outcomes for a particular care model. For example, a double-blinded RCT would likely indicate a high degree of confidence that the benefits outweigh the harms because of the rigorous process undertaken to reduce bias. On the other hand, a model of care with no pre-existing evidence based purely on scientific rationale might warrant a low degree of confidence. Where existing scientific evidence suggests that the harms outweigh the benefits, these innovative models of care should be rejected.

Next, evaluators should consider whether the innovative care model is within cost-effectiveness thresholds. While this may be difficult to assess if a model’s effectiveness is unknown, it may be possible to use a range of plausible estimates of cost and benefits.

Finally, the innovative model of care should be offered to patients who may validly consent or refuse and seek standard care or no care.

**Figure 2.**
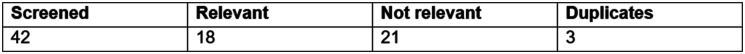
Papers reviewed for the purpose of this study.

In this paper, we apply our ethical framework for innovative care to two examples of allied health professionals offering care usually provided by ophthalmologists. First, we consider the example of nurses performing intravitreal injections. This practice is widely accepted as a safe and valid approach to expand the scope of practice for allied health practitioners in ophthalmological care. Second, we consider the case of optometrists expanding their practice to include a wider range of healthcare services beyond their current primary focus on eye examinations. This practice is less widely accepted (Figure 2).

## Methods

We conducted a literature review by identifying relevant articles on PubMed and Google Scholar through search terms. Articles from PubMed’s related article search were also included, and no limitations were imposed.

For PubMed, the search filter consisted of the following:

(((“Role of allied health personnel” OR “Allied health professionals”) AND (“decentralized eye care model” OR “community-based eye care” OR “primary eye care”)) AND (“ethical considerations” OR “ethics” OR “ethical implications” OR “quality of care” OR “training” OR “auditing”))

((allied health [Title/Abstract]) AND (decentralized [Title/Abstract])) AND (eye [Title/Abstract])

((allied health [Title/Abstract]) AND (decentralized [Title/Abstract])) AND (eye [Title/Abstract]) - ((allied health [Title/Abstract]) AND (decentralized care [Title/Abstract])) AND (eye [Title/Abstract])

((allied health [Title/Abstract]) AND (decentralized care [Title/Abstract])) AND (eye [Title/Abstract]) ((allied health [Title/Abstract]) AND (decentralized care model [Title/Abstract])) AND (eye [Title/Abstract])

((allied health [Title/Abstract]) AND (decentralized care model [Title/Abstract])) AND (eye [Title/Abstract])

For Google Scholar, the following search terms were implemented:

(“Role of allied health personnel” OR “Allied health professionals”) AND (“decentralized eye care model” OR “community-based eye care” OR “primary eye care”) AND (“ethical considerations” OR “ethics” OR “ethical implications” OR “quality of care” OR “training” OR “auditing”)

## Results

**Figure 3.**
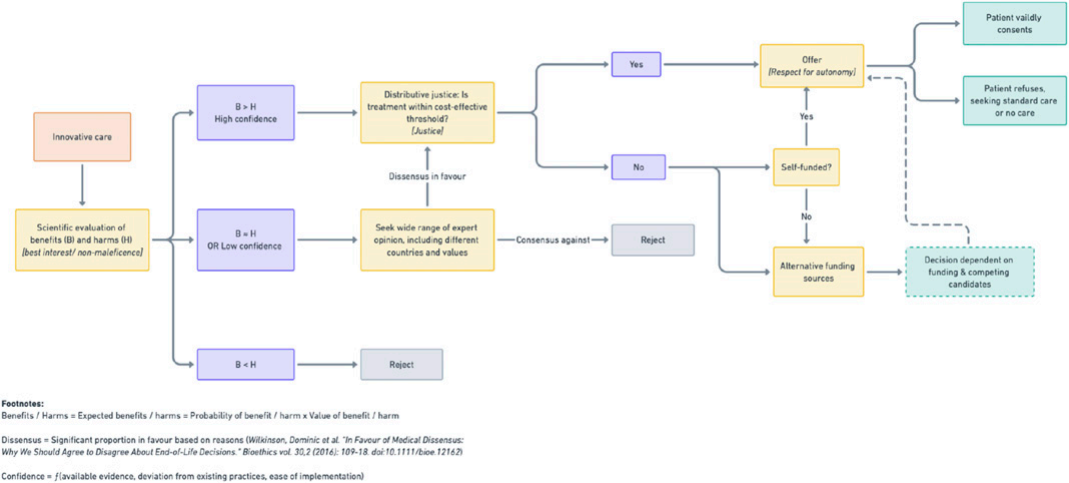
Innovative Care Algorithm for allied healthcare [adapted from Savulescu and Wilkinson, 2017].

A total of 42 papers were identified. These papers were then individually screened by members of the team independently and any discrepancies in decisions regarding the relevance of papers were raised at a discussion where a consensus was eventually met. Papers were included if they studied allied health personnel in the context of providing ophthalmological care for patients. We excluded papers that were incomplete (e.g., describing methodology), or of a different scale of care (e.g., healthcare systems). Of the 42 papers identified, there were 18 relevant papers, 21 papers deemed not relevant, and 3 duplicate entries that were excluded. We evaluate the results according to the Innovative Care Algorithm (Figure 3) and Four Principles of Biomedical Ethics: Respect for Autonomy, Beneficence, Non-maleficence, and Justice.

## Administration of intravitreal injections by nurses

### Reduced clinical delays leading to improved patient outcomes (Beneficence)

A retrospective audit of over 1000 intravitreal injections demonstrated that the waiting times for patients were reduced by almost half (from 29.5 to 15 days) when the service was nurse-led compared to performed by a doctor.^5^

A separate systematic literature review examining the role expansion of specialist ophthalmic nurses to deliver intravitreal injections also showed that this process offered timely delivery of services, and subsequently avoiding irreversible blindness in patients with wet age-related macular degeneration (AMD). There was an increase in patient satisfaction with nurse-led intravitreal injections in four out of the five studies included in the review and an absence of negative feedback.^6^

Nurse-led injections can increase capacity to deliver care. The number of intravitreal injections completed in a medical retina clinic over a 5-month window increased by 25% upon initiation of a nurse-directed intravitreal injection program as compared to a similar timeframe in the previous year.^7^ Additionally, a nurse-led service improved wait times and compliance to anti-vascular endothelial growth factor (VEGF) treatment guidelines, streamlining ophthalmology services and increasing patient satisfaction.^8^ Ultimately, prompt treatment facilitated by nurse-led services promotes the best interests of patients by reducing delays in service delivery and thereby reducing the risk of potential complications.

### Comparable safety profiles to ophthalmologist-led procedures (Non-maleficence)

A literature review of patients with wet AMD showed comparable clinical outcomes and complication rates when intravitreal injections were administered by trained ophthalmic nurses compared to ophthalmologist.^6^ Across various studies, nurse-led intravitreal injections resulted in very low rates of endophthalmitis, retinal detachment, vitreous hemorrhage, and other serious complications, demonstrating the safety and efficacy of nurse-led procedures.^5,7,9–12^

Adequate training is crucial to success. In Singapore, ophthalmic nurses undergo a rigorous training curriculum ensuring that they meet the competency requirements before they are allowed to administer intravitreal injections independently. This includes a minimum number of supervised procedures by a qualified ophthalmologist. They work closely with the ophthalmologists who decide on the clinical indication for treatment and provide readily available consult should complications or unexpected challenges arise.

The considerations of benefits (Beneficence) and risks (Non-maleficence) shows a clear balance of expected benefits over expected harms. Thus, according to the Innovative Care Algorithm, there should next be consideration of Justice (and whether the services are cost-effective).

### Enhanced accessibility to ophthalmologic services (Justice)

Nurse-led intravitreal injections are associated with significantly reduced cost per procedure for patients and institutions compared to doctor-led injections.^8^ This is equivalent to Singapore dollar (SGD) 4.6 and 6.6 million in potential cost savings annually for the Singapore National Eye Centre (SNEC) and Singapore, respectively.^12^ Reduced costs enable a greater number of patients (particularly financially disadvantaged individuals) access to eye care services.

Considerations of cost-effectiveness, and distributive justice, speak in favor of this form of allied healthcare. According to the Innovative Care Algorithm, it should be offered to patients as a part of publicly funded healthcare.

### Improved patient satisfaction (Respect for Autonomy)

Patient satisfaction was equivalent (if not superior) when intravitreal injections were administered by a nurse compared to a doctor.^6,7^ In particular, nurses scored higher than doctors in interpersonal manners and staff competency.^13^ Thus, offering patients the option of nurse-led services empowers them to make decisions about their care in line with their preferences. This respects autonomy and demonstrates such care can be offered to patients ethically.

## Challenges to nurse-led approaches

### Limitations due to legal regulations (Respect for Autonomy and Non-maleficence)

Regulations limit the scope of practice for nurses such that they are only legally permitted to administer certain licensed drugs. For example, nurses are currently not permitted to administer ranibizumab, an anti-VEGF injection used to treat acute macular degeneration. In order to expand the role of nurses to administer ranibizumab, healthcare institutions are required to seek legal counsel to ensure vicarious liability and verification of indemnity, as well as patients’ informed consent prior to the procedure.^7^

Applying the ethical framework for innovative care described above in Figure 3, this literature review provides robust scientific validation that the expected benefits of nurse-led intravitreal injections plausibly outweigh the potential harms. In particular, nurse-led care optimizes patient outcomes by reducing clinical delays and increasing access to ophthalmologic services, with comparable safety profiles to ophthalmologists. It also seems likely that nurse-led intravitreal injections will be cost-effective for healthcare institutions to deploy; however, this threshold may vary based on specific resources available to institutions. Given these considerations, nurse-led services may be offered to patients as an ethical form of innovative care. In time, nurse-led services may become the standard model of care for intravitreal injections, against which future variations in practice may be measured as a benchmark.

## Optometrists offering ophthalmological care

Expanding the role of optometrists to include tasks previously performed only by ophthalmologists could improve ophthalmologic care. However, this practice has yet to reach the same level of acceptance as nurse-led intravitreal injections.

### Improved patient outcomes (Beneficence)

Optometrists may be the first healthcare professionals to review patients with ocular signs of diseases like diabetes mellitus and have the potential to play a key role in detecting new eye diseases.^14^ While this is conventionally the role of ophthalmologists, optometrists do receive training in screening for ophthalmic diseases ranging from retinal disorders to glaucoma.^15^ Increasing the rates of screening by making such services more accessible to patients allows patients with ophthalmic diseases to be referred for further management in a timelier manner, avoiding complications associated with a delayed diagnosis.

Additionally, optometrists may assume the role of ophthalmologists in providing a first line service for eye care. Community Eye Clinics (CECs), established in Singapore and run by optometrists, demonstrated an increase in rates of subspecialty ophthalmology clinic referrals from 10.8% to 12.9% and ophthalmic interventions performed from 15.0% to 16.3%. These figures reflect ophthalmologists now having greater capacity to provide appropriate care for patients with more severe or urgent eye conditions as a result of optometrists in CECs taking on the role of managing patients with more stable eye conditions.^16^ As such, expanding the role of optometrists may improve patient outcomes by more appropriately matching patients to healthcare professionals according to the severity and level of care required for their needs.

### Increase access to ophthalmologic care (Justice)

Optometrists enable greater availability of screening than conventionally provided by ophthalmologists. For example, collaborating with optometrists in rural areas led to an increase in Aboriginals having eye examinations.^17^ Optometrists were also shown to have the capacity to communicate with ophthalmologists over satellite link in remote areas of Tamil Nadu to receive guidance on further treatments and investigations needed.^18^

In another example, community eye clinics have been explored in Singapore as an option to improve the accessibility of eye care services. They have been demonstrated to reduce first visit referrals from one polyclinic to the ophthalmology clinic by 27.5%.^16^ Involving optometrists in the eye care model serves to overcome barriers like physical distance that may render the existing system inaccessible to certain patients. With an aging population seen in many countries and the increase in the demand of eye care, allowing the optometrist to take over the role of eye screening and monitoring of stable eye conditions will allow the ophthalmologist to focus on the management of acute and more complex eye conditions.

### Cost-efficient and timesaving (Distributive Justice)

Involvement of optometrists in performing selective laser trabeculoplasty (SLT) is viewed as beneficial for the United Kingdom’s National Health Service (NHS) in multiple ways including cost, and patients are receptive to this proposal—optometrist-led SLT in the NHS was perceived to be cost saving due to remuneration grade differences.^19^ Optometrist-SLT would increase capacity and provide greater continuity of care for patients, especially in the near future. Optometrist-delivered SLT would lead to consultants having more capacity to attend to complex cases, and this is vital since backlogs are expected to increase with around 30% of senior ophthalmologists in the United Kingdom reaching retirement but around 20% of the workload in the current glaucoma service requires a specialist, with the number of glaucoma patients projected to increase in an aging population.^19^

There also exist a significant proportion of patients who would be more suited to outpatient care. The capacity of allied health professionals may be utilized to create cost savings. An audit comparing standard hospital care and a collaborative care model showed that among 65 low-risk referrals made to the latter, 68.2% of the patients did not need hospital appointments. Costs incurred were 43% less than that of hospital care.^20^

Another audit comparing standard hospital care and a collaborative care model showed that among 321 low-risk referrals made to the latter, 57% of the patients did not require hospital visits and costs incurred were 22% less than that of hospital care.^21^

With regards to the competency of optometrists for such a, a study comparing decision-making of ophthalmologists and optometrists required participants to have a minimal level of competency and demonstrated the potential for optometrist-led monitoring services to reduce the patient load for ophthalmologists. Included in the study was the optometrists’ assessment which was shown to not be inferior to ophthalmologists’ (84.4% and 85.4% correct assessments for optometrists and ophthalmologists, respectively).^22^ If optometrists can demonstrate a level of competency that meets accepted standards, they will be able to share a proportion of the patient load faced by ophthalmologists and allow allocation of healthcare resources that is more equitable.

## Challenges to expanded optometrist care

### Higher rates of complications and poorer clinical outcomes (Non-maleficence)

Optometrist-led procedures faced higher rates of complications and poorer outcomes as compared to those performed by ophthalmologists, making such an expansion possible run counter to the principle of Non-maleficence. Patients who received SLT by an optometrist were more likely to require subsequent SLT session as compared to an ophthalmologist. There was a 189% increased probability of requiring additional SLT in the same eye in patients who underwent SLT by optometrists as compared to ophthalmologists.^18^

Optometrists were shown to have higher rates of delayed retinal tears and detachment among patients with posterior vitreous detachment than ophthalmologists. Incidence rates of delayed breaks and detachments were 1.8% (5/282) and 0.7% (2/282), respectively, in the retina attending group, 1.0% (1/105) and 1.0% (1/105) in the non-retina ophthalmology attending group, 4.7% (3/64) and 0% (0/64) in the optometrist group, and 2.5% (1/40) and 0% (0/40) in the ophthalmology resident only group. While one study showed there was no statistically significant difference in the incidence of delayed break or detachment among the staffing groups (*p* = .7312), this study was underpowered to detect a statistically significant difference among staffing groups.^23^

### Increased difficulties in diagnosis and screening (Non-maleficence)

Optometrists face difficulties in diagnosing and screening due to the extension of their role beyond their training and equipment. Common examples include a survey where not all optometrists surveyed regarding keratoconus diagnosis had access to the full range of ophthalmologic equipment, with only 56% of them had access to a keratometer and 86% having access to a slit lamp biomicroscope, equipment that are typically used in keratoconus diagnosis^24,25^ Consequently, inadequate facilities was the top reason that surveyed optometrists from a public hospital referred keratoconus patients to their colleagues in private practice, with 60% of them responding that they lacked a keratometer.^24,25^ None of the facilities had equipment to fit contact lenses, resulting in the inability to provide for the management of these patients.^24,25^

There is a need for education and guidelines requiring use of validated screening tools to ensure minimum standard of care will be provided by allied health staff, with a study showing that while 81% of optometrists surveyed often asked patients about migraine symptoms, almost all of them (92%) did not utilize any validated migraine screening tools.^26^ It could be premature at this point to involve certain groups of healthcare professionals in specialized roles without adequate preparation.

### Less preferred among patients (Respect for Autonomy)

A study showed a preference of patients for screening conducted by general practitioners instead of optometrists. In fact, 15.7% of the study group reported considering screening elsewhere, for example, general practitioners and pharmacists.^27^ This could play a role in the effectiveness of a novel method of eye care delivery involving healthcare professionals holding specialized roles and the need for patient education.

With reference to our Innovative Care Algorithm (Figure 3), the proposed model of care to expand optometrists’ functioning as physician extenders is scientifically plausible and cost-effective. However, from the studies above, it is unclear whether this model of care is expected to present a clear benefit to patients over its potential harms. In particular, patients may be at greater risk of experiencing complications or poorer clinical outcomes, or they may prefer a different healthcare professional. In this case, it may be appropriate to seek a wide range of expert opinion on the appropriateness of a particular role for optometrists as physician extenders. If there is reasonable disagreement (i.e., dissensus) among experts in favor of the expected benefits outweighing the harms, then it may be appropriate to offer patients this model of care (provided that it is within cost-effective thresholds).

In addition, the proposed ethical framework showed the gaps and areas of weakness in the new model of care which should be addressed, particularly in the area of training and equipment. In the case of optometrists being physician extenders, the main gaps include the standardized training and accreditation; equipping optometrists’ practices with appropriate diagnostic devices; and improving patient/public awareness on the roles of trained optometrists in eye care.

## Discussion and future research directions

Given the many examples of the benefits that optometrists and nurses can bring to eye health, it would be prudent to explore the synergistic collaboration between the two of them. By playing to each groups’ strengths, we can achieve even greater efficiency and reach of eye health provision. Some examples may include having outpatient centers where optometrists with more specialized training in diagnosing ophthalmic conditions can take on the role of screening, whereas nurses who have their training more oriented towards procedures than optometrists can take on the role of administering medications like intravitreal injections.

An aspect of ophthalmological care not well studied but with a definite impact on the outcomes would be the influence of profit-driven corporations. Doctors, nurses, and especially optometrists in private practice often practice under large for-profit corporations and organizations. It is essential that there is no conflict of interest and that care follows the pathway of the Innovative Care Algorithm, not one aimed solely at increased profit.

There is potential for future studies to explore the reality of commercial interests and its prevalence in the provision of healthcare (for the purposes of this study, in the area of optometry). Future studies may also find relevance in describing the relationship between allied health providers (e.g., optometrists) deferring to commercial interests and patient outcomes. In so doing, one may also evaluate if practices differ between private and public health as a result of them. Findings may suggest a need to focus on placing safeguards to ensure profit-driven motivations do not compromise patient care.

One limitation of the current study stems from its nature as a review of existing literature which did not focus on patient experience. It would be meaningful to compare patient experiences, whether the initial contact is with an allied health member who referred the patient to an ophthalmologist, or an ophthalmologist who refers the patient to an allied health member to assist in the treatment process. Assessing this temporal relationship and sequence of events could give insights into the patient’s complete journey through the healthcare system.

## Conclusion

The expanding roles of allied health personnel in healthcare in general and in ophthalmology in particular are an eventuality given the trajectory of demand for care. It also prompts the inevitable question of its acceptability to stakeholders and the need for analysis through established ethical principles. While there are important successes, it is important not to neglect the risks highlighted through our comparison as well. Several risks remain with innovative allied healthcare. These include a lack of training and equipment that suitably prepares allied health staff for role expansion and legislation that lags behind in the face of a rapidly evolving medical landscape. Through this paper, we hope future steps can be taken to modernize and improve on care utilizing allied healthcare professionals and innovate further to achieve optimum outcomes while remaining grounded by ethical principles.

## References

[1] Wilkinson D Savulescu J . After Charlie Gard: ethically ensuring access to innovative treatment. Lancet 2017; 390(10094): 540–542. 28792386 10.1016/S0140-6736(17)32125-6

[2] McCulloch P Altman DG Campbell WB , et al.. No surgical innovation without evaluation: the IDEAL recommendations. Lancet 2009; 374(9695): 1105–1112. 19782876 10.1016/S0140-6736(09)61116-8

[3] Hutchison K Rogers W Eyers A , et al. Getting clearer about surgical innovation: a new definition and a new tool to support responsible practice. Ann Surg 2015; 262(6): 949–954. 25719812 10.1097/SLA.0000000000001174

[4] Afnan MAM Liu Y Conitzer V , et al. Interpretable, not black-box, artificial intelligence should be used for embryo selection. Hum Reprod Open 2021; 2021(4): hoab040. 34938903 10.1093/hropen/hoab040 PMC8687137

[5] Raman V Triggol A Cudrnak T , et al. Safety of nurse-led intravitreal injection of dexamethasone (Ozurdex) implant service. Audit of first 1000 cases. Eye 2021; 35(2): 388–392. 32728227 10.1038/s41433-020-1114-7 PMC8027221

[6] Gregg E . Nurse-led ranibizumab intravitreal injections in wet age-related macular degeneration: a literature review. Nurs Stand 2017; 31(33): 44–52. 10.7748/ns.2017.e10344 28399772

[7] DaCosta J Hamilton R Nago J , et al. Implementation of a nurse-delivered intravitreal injection service. Eye 2014; 28(6): 734–740. 24699166 10.1038/eye.2014.69 PMC4058629

[8] Michelotti MM Abugreen S Kelly SP , et al. Transformational change: nurses substituting for ophthalmologists for intravitreal injections - a quality-improvement report. Clin Ophthalmol 2014; 8: 755–761. 24790403 10.2147/OPTH.S59982 PMC3998867

[9] Ahmed I Maghsoudlou P Hasan H , et al. Safety and efficacy of nurse led intravitreal injection service with Precivia® injection assist device. Eur J Ophthalmol 2022; 32(5): 2771–2776. 34791908 10.1177/11206721211060947

[10] Simcock P Kingett B Mann N , et al. A safety audit of the first 10 000 intravitreal ranibizumab injections performed by nurse practitioners. Eye 2014; 28(10): 1161–1164. 25033899 10.1038/eye.2014.153 PMC4194327

[11] Rasul A Subhi Y Sørensen TL , et al. Non-physician delivered intravitreal injection service is feasible and safe - a systematic review. Dan Med J 2016; 63(5): A5229. 27127016

[12] Teo AWJ Rim TH Wong CW , et al. Design, implementation, and evaluation of a nurse-led intravitreal injection programme for retinal diseases in Singapore. Eye 2020; 34(11): 2123–2130. 32382144 10.1038/s41433-020-0920-2 PMC7784933

[13] Silpa-Archa S Limwattanayingyong J Tadarati M , et al. Capacity building in screening and treatment of diabetic retinopathy in Asia-Pacific region. Indian J Ophthalmol 2021; 69(11): 2959–2967. 34708730 10.4103/ijo.IJO_1075_21 PMC8725108

[14] Eye care of the patient with diabetes mellitus 2nd ed. Missouri: American Optometric Association, 2019.

[15] Barnard NA . Screening by optometrists. Ophthalmic Physiol Opt 1983; 3(3): 365–368. 6646773

[16] Yunqi K Kelvin LZ Lian YS , et al. Impact of community eye clinics (CEC) on specialist eye clinic referrals. Ophthalmic Epidemiol 2024; 31(4): 315–320. 37817451 10.1080/09286586.2023.2261528

[17] Main R . Issues pertaining to recruitment and retention of rural and remote optometrists in Australia. Sydney: UNSW, 2012.

[18] Verma M Raman R Mohan RE . Application of tele-ophthalmology in remote diagnosis and management of adnexal and orbital diseases. Indian J Ophthalmol 2009; 57(5): 381–384. 19700877 10.4103/0301-4738.55078 PMC2804127

[19] Konstantakopoulou E Jones L Nathwani N , et al. Selective laser trabeculoplasty (SLT) performed by optometrists-enablers and barriers to a shift in service delivery. Eye 2022; 36(10): 2006–2012. 34389819 10.1038/s41433-021-01746-0 PMC8362647

[20] Tahhan N Ford BK Angell B , et al. Evaluating the cost and wait-times of a task-sharing model of care for diabetic eye care: a case study from Australia. BMJ Open 2020; 10(10): e036842. 10.1136/bmjopen-2020-036842 PMC7537459 33020087

[21] Ford BK Angell B Liew G , et al. Improving patient access and reducing costs for glaucoma with integrated hospital and community care: a case study from Australia. Int J Integrated Care 2019; 19(4): 5. 10.5334/ijic.4642 PMC6838764 31749669

[22] Reeves BC Scott LJ Taylor J , et al. The Effectiveness, cost-effectiveness and acceptability of Community versus Hospital Eye Service follow-up for patients with neovascular age-related macular degeneration with quiescent disease (ECHoES): a virtual randomised balanced incomplete block trial. Health Technol Assess 2016; 20(80): 1–120. 10.3310/hta20800 PMC5107885 27809956

[23] Patel SN Lee C Cui D , et al. Association of staffing with Incidence of delayed retinal break or detachment after posterior vitreous detachment in a resident urgent care clinic. Graefes Arch Clin Exp Ophthalmol 2022; 260(3): 791–798. 34661735 10.1007/s00417-021-05437-0

[24] Gcabashe N Moodley VR Hansraj R . Keratoconus management at public sector facilities in KwaZulu-Natal, South Africa: practitioner perspectives. African Vision and Eye Health 2022; 81(1): 698.

[25] Nkoana PM Moodley VR Mashige KP . Self-reported knowledge and skills related to diagnosis and management of keratoconus among public sector optometrists in the Limpopo province, South Africa. Afr J Prim Health Care Fam Med 2022; 14(1): e1–e9. 10.4102/phcfm.v14i1.3668 PMC9772765 36546489

[26] Nguyen BN Singh S Downie LE , et al. Migraine screening in primary eye care practice: current behaviors and the impact of clinician education. Headache 2020; 60(8): 1817–1829. 32767768 10.1111/head.13920

[27] Howse J . Helen, Screening for diabetes in optometric practice. Durham theses: Durham University, 2010.

